# Contrast reversal of the iris and sclera increases the face sensitive N170

**DOI:** 10.3389/fnhum.2022.987217

**Published:** 2022-09-07

**Authors:** Kelly J. Jantzen, Nicole McNamara, Adam Harris, Anna Schubert, Michael Brooks, Matthew Seifert, Lawrence A. Symons

**Affiliations:** Department of Psychology, Western Washington University, Bellingham, WA, United States

**Keywords:** N170, eyes, face, contrast, inversion

## Abstract

Previous research has demonstrated that reversing the contrast of the eye region, which includes the eyebrows, affects the N170 ERP. To selectively assess the impact of just the eyes, the present study evaluated the N170 in response to reversing contrast polarity of just the iris and sclera in upright and inverted face stimuli. Contrast reversal of the eyes increased the amplitude of the N170 for upright faces, but not for inverted faces, suggesting that the contrast of eyes is an important contributor to the N170 ERP.

## Introduction

Faces arguably represent a distinct class of stimuli that are processed by specialized distributed neural circuits (e.g., McKone and Robbins, 2011). Inverting face stimuli by 180 degrees disrupts their encoding and discriminability (Freire et al., 2000), more than other objects (Yovel and Kanwisher, 2005). Reversing the contrast of face stimuli (Nederhouser et al., 2007) has similar effects and both inversion and contrast reversal effects are often cited as evidence for the specialized processing of faces. Both face inversion (Bentin et al., 1996; Eimer, 2000; Rossion et al., 2000; Taylor et al., 2001; de Haan et al., 2002; Itier and Taylor, 2002, 2004a,b; Itier et al., 2006a) and contrast reversal (Itier and Taylor, 2002, 2004b) increase the latency and amplitude of the face sensitive N170 component of the electroencephalograph (EEG) and its magnetic counterpart (magnetoencephalograph) (Itier et al., 2006a,b), both of which are signatures of early cortical processing that likely represent structural encoding of faces (Rossion et al., 1999; Eimer, 2000).

The eyes play a crucial role in face processing, which suggests that the eyes undergo neural processing distinct from more general face processing (Nemrodov and Itier, 2011; Zheng et al., 2011). For example, observing eyes alone elicits an N170 greater in amplitude than observing a whole face (Bentin et al., 1996). To specifically test the importance of the eyes in the configural processing of faces, Itier et al. (2007) recorded the N170 in response to viewing faces, faces without eyes, and eyes without faces. They found that, for inverted face stimuli, eyes alone produced a similar N170 to faces with eyes, both of which were larger than for faces without eyes. Further, inversion and contrast reversal of the faces affected the N170 only in the presence of eyes leading to the conclusion that configural disruption of a face is driven largely by the eyes.

The hypothesized importance of the eyes is in keeping with behavioral data demonstrating that eyes are at the core of facial recognition, emotion evaluation, and directing shared attention (reviewed by Itier and Batty, 2009). For instance, an observer’s attention is directed by their perceived direction of gaze, which is determined, in part, by the brightness of the sclera on each side of the iris (Ando, 2004). The direction of perceived gaze also affects the N170, which is increased when gaze is directed at the viewer (e.g., Conty et al., 2007; Burra et al., 2017) and when it is dynamically averted (Latinus et al., 2015; Rossi et al., 2015). Reversing contrast of just the eyes disrupts the ability to determine gaze direction (Ricciardelli et al., 2000; Olk et al., 2008) and may account for previous findings. However, the contrast reversed stimuli of Itier et al. (2007) included a region beyond the eyes alone including, for example, the forehead, the bridge of the nose and the eyebrows. Watt et al. (2007) found that the eyebrows could interfere with the perception of gaze, and Sadr et al. (2003) demonstrated that eyebrows were equally influential as eyes for recognizing faces. This highlights the potential importance of other localized features or combinations of features in driving the effects of contrast reversal and inversion.

We tested the specific hypothesis that the critical importance of the eyes in face processing comes, at least partly, from the information gleaned from the contrast between the sclera and pupil. We measured the N170 from participants while presenting upright and inverted faces and cars with eyes (or headlights) that were either normal or contrast reversed. Importantly, contrast reversal was restricted to the sclera and pupil of the eye so that any changes in N170 amplitude would be attributable specifically to the eye itself, not other local features. We also assessed the interaction of inversion of face images with contrast reversal of the eye region. A disruption of configural processing by contrast reversal of the eye region should result in a larger N170 for contrast reversed eye stimuli than normal eye stimuli only when upright since configural processing is disrupted in face stimuli during inversion (Freire et al., 2000; however, see Schwaninger et al., 2005). It was predicted there would be no effects of inversion or contrast reversal on the N170 for the control car stimuli (Kloth et al., 2013).

## Methods

### Participants

Data were collected from 18 participants with normal to corrected vision. Data from two participants was removed due to excessive eye artifact and poor behavioral performance (<85% correct) leaving 16 participants (8 females) with a mean age of 23.5 (*SD* = 8.1). All participants were provided informed consent for this research which was authorized by the internal review board of Western Washington University.

### Stimuli

Fifty standardized White faces (25 female) were selected from the Chicago Face Database 2.0 (Ma et al., 2015). Faces were all full color, front facing adults with a neutral expression. Using WebMorph (DeBruine and Tiddeman, 2016), all face images were standardized in size, scale, and position. Faces were individually delineated by manually placing specific coordinates over each image’s facial landmarks (i.e., hairline, jawline, eyes) to standardize pupil level and face size. The outline of the face, defined by the jawline and hairline, was used to mask the ears, hair, and neck so that only the face of each image was visible against a white background. Images were scaled so that the face was approximately 800 × 1,200 pixels (9.1 × 13.6 visual angle) and converted to grayscale by eliminating hue and saturation information while maintaining luminance. To account for differences in contrast among images, pixel values were adjusted such that the mean and standard deviation of all non-background pixels was equivalent across images.

Control stimuli were 50 white/silver cars collected online from manufacturer websites. They were standardized in appearance so that the car was centered in the image, and turned slightly to the right, displayed on a white background, and sized 600 × 800 pixels. Contrast was adjusted in a manner similar to the faces.

**FIGURE 1:**
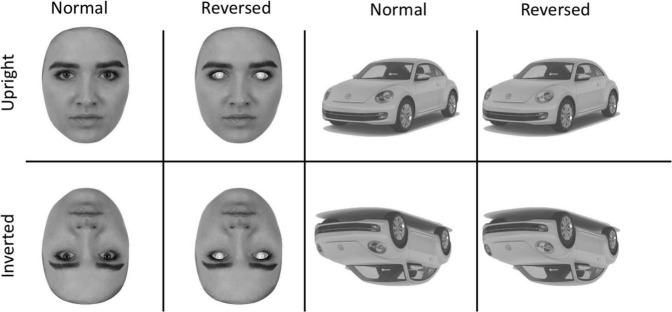
Sample Stimuli showing the upright **(top)** and inverted **(bottom)** versions of the faces and cars The second column shows faces with eye contrast reversed and the fourth column shows the car with the headlights reversed.

Contrast reversed versions of all images were created in Adobe Photoshop using the “Invert” function. For faces, contrast reversal was applied to pixels making up the sclera, iris, and pupil of the eyes. For cars, contrast reversal was applied to pixels making up the headlights. Inverted versions of the normal and contrast reversed stimuli were created by rotating the images 180 degrees around the image center. The procedures resulted in the generation of 50 stimuli in each of 8 experimental conditions defined by the factors of Stimulus (Faces/Cars), Orientation (Upright/Inverted), and Contrast (Normal/Reversed). Sample face and car images are in Figure 1.

### Task

The stimuli were presented on a 19-inch Dell LCD monitor using Inquisit 5.0. On each trial, participants were presented with a fixation cross, followed by a stimulus from one of the eight experimental conditions for 250 ms. To reduce the possible, confound of gaze fixation (e.g., Nemrodov et al., 2014; Fisher et al., 2016) participants gaze was directed to the eyes using a fixation cross that appeared in the horizontal midpoint of the image and was vertically aligned with the middle of the pupils. For cars, the fixation cross was in the center of the image. Participants used their dominant hand to press a key to indicate whether the stimulus presented was a face or a car. A trial ended when the participant responded or after 1,500 ms. Trials were separated by a random interval ranging from 1,000 to 1,300 ms. A block of trials was completed when all the 400 stimuli had been presented. Participants completed 2 blocks for a total of 100 trials per condition.

### Electroencephalograph recording and processing

A BioSemi ActiveTwo (Biosemi, Amsterdam, Netherlands) was used to continuously sample EEG (512 Hz) from 64 scalp electrodes placed according to the 10-5 electrode system (Oostenveld and Praamstra, 2001). EEG signals were processed in Matlab 9.8 (Mathworks Inc., Natick, MA, United States) using a combination of in-house routines and the EEGlab toolbox (Delorme and Makeig, 2004). Signals were pass filtered above 0.1 Hz. Bad channels were replaced by spherical interpolation of the remaining channels before average referencing. Line noise was reduced using the EEGLab plugin CleanLine, which adaptively estimated and removed sinusoidal noise at 60 Hz. Individual EEG epochs from −100 to 500 ms around the onset of the stimulus were extracted for each trial on which the participant correctly identified the stimulus as either a face or car (95 ± 3% of trials).

### Data analysis

After rejecting trials in which peak-to-peak voltage exceeded 400 uV (see Supplementary Table 2 for the mean number of trials in each condition), we decomposed signals into a set of independent components (Jung et al., 2001) and classified the source of each component using the ICLabel plugin for EEGLab. To remove artifacts from the data (Jung et al., 2001), we subtracted components confidently identified (>60%) as eye-blinks, muscle artifacts, heartbeat, line-noise or channel-noise. The cleaned trials were then low pass filtered below 50 Hz before averaging.

**FIGURE 2:**
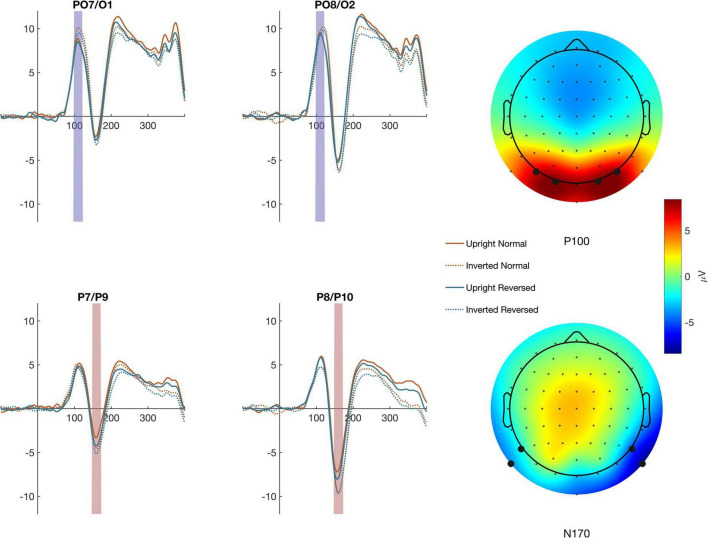
Averages across the channel montages used to define the P100 **(top traces)** and N170 **(bottom traces)** are shown for each condition The colored regions highlight the timepoints used to calculate ERP amplitude. The mean activity across the time windows is shown in the topographic maps to the right.

ERP amplitudes were defined as the average within a time window and across channels selected based on scalp topographies described in the literature and after confirmation that the selected channels demonstrated the largest amplitudes in each hemisphere (see Figure 2). The N170 was defined between 150 and 180 ms in P7/P9 (left) and P8/P10 (right). To determine if N170 effects may have resulted from changes in the low-level stimulus features evaluated earlier in cortical processing, we also analyzed stimulus related changes in P100 amplitude. The P100 was evaluated between 90 and 130 ms in at electrodes PO7/O1 (left)and PO8/O2 (right) (see Figure 2). ERP amplitudes were subjected to separate within subject ANOVAs.

## Results

### P100

**FIGURE 3:**
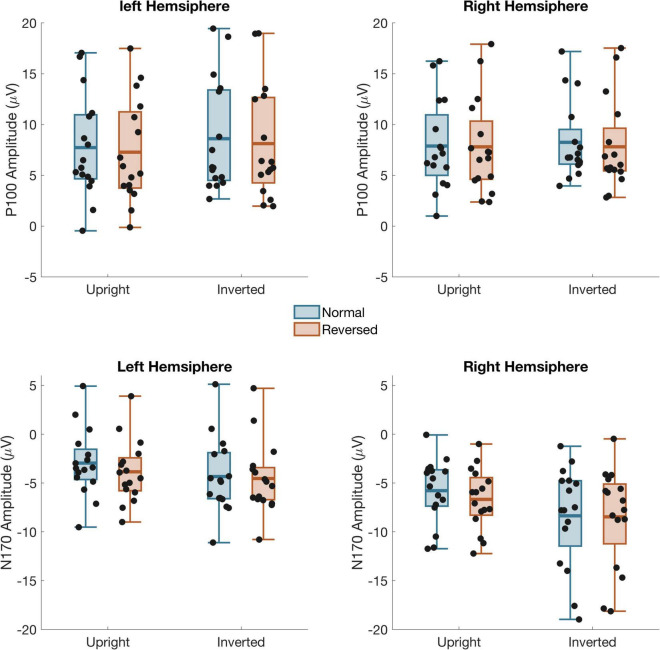
Mean amplitude from left and right hemisphere channel groups for the P100 **(top)** and N170 **(bottom)** time windows Black dots: individual subject data. Box shows the mean and first and third quartile. The whiskers show the 2nd to 9th quantile.

P100 amplitude (Figure 3) was evaluated using a within-subject ANOVA with factors Contrast (Normal/Reversed), Orientation (Upright/Inverted), Stimulus (Face/Car), and Hemisphere (Left/Right). There were no significant effects of Contrast, Orientation, Stimulus or Hemisphere and no significant interaction, indicating that our manipulations did not affect early stage of visual processing. See full ANOVA results in Supplementary Table 1.

### N170

A 4-way ANOVA on N170 amplitude (Figure 3) found a main effect of Contrast, *F*_(1,15)_ = 5.81, *p* = 0.029, Orientation, *F*_(1,15)_ = 12.7, *p* = 0.003, Stimulus, *F*_(1,15)_ = 38.1, *p* < 0.001, and Hemisphere, *F*_(1,15)_ = 13.9, *p* = 0.002. There was also a two-way interaction between Orientation and Stimulus, *F*_(1,15)_ = 10.3, *p* = 0.006, and three way interaction between Contrast, Orientation, and Stimulus *F*_(1,15)_ = 5.49, *p* = 0.033 as well as Orientation, Stimulus, and Channel, *F*_(1,15)_ = 8.72, *p* = 0.010. Separate three-way ANOVA’s on the Cars and Faces showed that the interactions with Stimulus occurred because the N170 was unaffected by Inversion or Contrast Reversal of the cars. Although the N170 in response to Cars was larger in the right than left hemisphere, *F*_(1,15)_ = 11.6, *p* = 0.004, it was unaffected by Inversion or Contrast reversal. For Faces, the N170 was also larger in the right hemisphere, *F*_(1,15)_ = 13.1, *p* = 0.002. Unlike the response to cars, however, the N170 to faces was significantly larger when faces were inverted, *F*_(1,15)_ = 23.4, *p* < 0.001, and when the eyes were contrast reversed, *F*_(1,15)_ = 6.45, *p* = 0.023. Critical to our predictions, there was also a significant interaction between Contrast and Orientation, *F*_(1,15)_ = 4.62, *p* = 0.048, such that the N170 was larger for reversed eyes than normal eyes when faces were upright [*F*_(1,15)_ = 8.82, *p* = 0.009], but not inverted [*F*_(1,15)_ = 0.45 *p* = 0.51].

## Discussion

Consistent with studies in which a broader eye region, including eyebrows, parts of the forehead, and the bridge of the nose, was manipulated (Itier et al., 2007; Fisher et al., 2016), contrast reversal of the eyes alone increased the amplitude of the early face-sensitive N170—a difference that was eliminated under face inversion. Enhancement of the N170 component to faces with contrast-reversed eyes has been interpreted as a marker for impaired structural encoding of a face processing mechanism that is tuned or biased toward the eye region (Fisher et al., 2016) and is cited as support for the existence of eye specific processing in humans (e.g., Itier and Batty, 2009). Our results further suggest that previous findings may be accounted for, at least partly, by a disruption of the white/dark contrast of the eye itself. The finding that face inversion eliminated N170 amplitude differences corroborates the conclusion that the seemingly “within feature” contrast reversal of the eye alone is sufficient to disrupt structural encoding of a face since the same effects were not observed when face configuration was disrupted by inversion.

The white sclera in human eyes facilitates the perception of gaze direction (Kobayashi and Kohshima, 1997, 2001; Ando, 2004), which supports social interaction by indicating shared attention and enabling inferences of others’ mental state (e.g., Stephenson et al., 2021). Although the specific impact of eye gaze direction on the N170 is inconsistent and may be task and context dependent (Hadders-Algra, 2022), evidence supports the general conclusion that information about gaze direction is extracted early in face processing. Burra et al. (2017) and others (see Hadders-Algra, 2022 for a recent review) reported an increase in N170 amplitude in response to briefly presented faces depicting direct compared to averted gaze suggesting that face processing is sensitive to gaze direction. Rossi et al. (2015) further showed a larger N170 in response to dynamic shifts of gaze away from compared to toward the viewer, for real but not line-drawn faces, suggesting that the N170 is sensitive to the low-level light-dark contrast. Our current findings provide support for this general hypothesis and suggest that due to its important social value, the ability to decode gaze direction from the sclera/iris contrast may be a central component in face processing and in categorizing an object as a face.

Although our results show the impact of altering eye contrast on early cortical processing, recent findings suggest that this effect may be context dependent. For instance, Latinus et al. (2015) found that social context may enhance the salience of gaze processing via top-down effects of the brain operating in a “socially aware” mode. More recent work points to the importance of the N170 in the establishment of shared attention. Indeed, Stephenson et al. (2020) found an enhanced N170 when changes in eye gaze of a stimulus face were in the same direction as the participant, indicating the establishment of shared attention. In both these studies, N170 responses were measured in response to gaze shifts, highlighting that an N170 can be elicited not only by eyes alone, but by a change in the relative contrast between sclera and iris.

The present work suggests that the effects reported by Itier et al. (2007) are driven exclusively by the eyes, and that eyebrows are less important than would be predicted based on behavioral findings (Watt et al., 2007). Nonetheless, eyebrows are a high contrast feature effective in communicating emotion and non-verbal context cues, which raises questions concerning their ability to influence early face processing measured by the N170. A better understanding of the relative and additive contribution of eyes and eyebrows may be gained by manipulating both features independently within the same experimental context.

In summary, this study demonstrated that faces with contrast-reversed sclera/iris generate a larger N170 when presented upright but not inverted—a signature of disrupted structural encoding. Beyond further crystallizing the privileged position of the eyes in face processing, this work shows that the local contrast between the light sclera and dark iris and pupil may be the central component of what makes the eyes so important for face processing. Although a relatively localized feature, our results indicate that contrast reversed eyes disrupt early face processing suggesting that the natural contrast present in human eyes aids in characterizing an object as a face.

## Data availability statement

The raw data supporting the conclusions of this article will be made available by the authors, without undue reservation.

## Ethics statement

The studies involving human participants were reviewed and approved by the Western Washington University Institutional Review Board. The patients/participants provided their written informed consent to participate in this study.

## Author contributions

KJ provided lab equipment and programming, contributed to data analysis, wrote first draft of manuscript, and edited subsequent and final drafts. LS provided original concept of research and wrote and edited subsequent and final drafts of manuscript. NM, AH, and AS worked on early conceptualizations of research idea, created stimuli, collected data, and provided feedback on drafts of manuscript. MB edited and wrote drafts of the manuscript. MS worked on early conceptualizations of research idea and edited and wrote drafts of the manuscript. All authors contributed to the article and approved the submitted version.
