# Supplementary material for: Contrast reversal of the iris and sclera increases the face sensitive N170
Source: Front Hum Neurosci. 2022 Sep 7;16:987217. doi: 10.3389/fnhum.2022.987217 (PMC9491205; doi:10.3389/fnhum.2022.987217)
Supplement: Supplementary file 1 [file Table_1.docx]

| Supplementary Table 1. Statistical results from the P100 Analysis of Variance | | |
| --- | --- | --- |
| Effect | *F* | *p* |
| Contrast | 2.60 | 0.128 |
| Orientation | 0.55 | 0.470 |
| Stimulus | 0.31 | 0.588 |
| Channel | 0.25 | 0.625 |
| Orientation * Contrast | 0.01 | 0.929 |
| Orientation * Stimulus | 1.60 | 0.226 |
| Contrast * Stimulus | 3.42 | 0.084 |
| Orientation * Channel | 0.91 | 0.355 |
| Contrast * Channel | 4.12 | 0.061 |
| Stimulus * Channel | 2.21 | 0.158 |
| Orientation * Contrast * Stimulus | 0.39 | 0.544 |
| Orientation * Contrast * Channel | 0.22 | 0.643 |
| Orientation * Stimulus * Channel | 0.03 | 0.873 |
| Contrast * Stimulus * Channel | 0.01 | 0.911 |
| Orientation * Contrast * Stimulus * Channel | 0.19 | 0.669 |
