# Supplementary material for: Contrast reversal of the iris and sclera increases the face sensitive N170
Source: Front Hum Neurosci. 2022 Sep 7;16:987217. doi: 10.3389/fnhum.2022.987217 (PMC9491205; doi:10.3389/fnhum.2022.987217)
Supplement: Supplementary file 2 [file Table_2.docx]

| Supplementary Table 2. The mean and standard deviation for number of trials in each experimental condition. | | | |
| --- | --- | --- | --- |
| Condition | Mean | SD |  |
| UNF | 95.9 | 3.9 |  |
| URF | 95.3 | 3.8 |  |
| INF | 95.9 | 2.9 |  |
| IRF | 94.6 | 4.1 |  |
| UNC | 94.4 | 4.4 |  |
| URC | 93.8 | 3.9 |  |
| INC | 96.4 | 3.5 |  |
| IRC | 95.4 | 3.8 |  |
| U = Upright orientation, I = Inverted orientation; N = Normal contrast; R = Reversed contrast; F = Faces, C = Cars | | | |
